# Decoding the Drivers of Consumer Choice for Near-Expired Meat in Digital Retailing: Economic Incentives, Food Safety, and Technology Acceptance

**DOI:** 10.3390/foods15152680

**Published:** 2026-07-29

**Authors:** Wen-Shin Huang, Cai-Wei Lin, Kuang-Ling Lai, Han-Shen Chen

**Affiliations:** 1 Department of Business Administration, Chaoyang University of Technology, Taichung 413310, Taiwan; wshuang@cyut.edu.tw (W.-S.H.); lcwtina0522@gmail.com (C.-W.L.); 2 Department of Applied Economics, National Chung Hsing University, Taichung 40227, Taiwan; ds178294@gmail.com; 3 Department of Health Industry Technology Management, Chung Shan Medical University, Taichung 40201, Taiwan; 4 Department of Medical Management, Chung Shan Medical University Hospital, Taichung 40201, Taiwan

**Keywords:** near-expired meat, food choice behavior, food safety, price discount, digital food retail, sustainable consumption

## Abstract

This study investigates consumers’ intentions to purchase high-risk, near-expired fresh meat via a supermarket mobile app, addressing food waste in a digital retail context. An integrated framework combining the technology acceptance model and the theory of planned behavior was employed, incorporating price discounts and food and environmental safety concerns. Data from 418 users of Taiwan’s leading supermarket chain were analyzed using partial least squares structural equation modeling (PLS-SEM). The findings reveal that perceived usefulness strongly drives app usage attitude while environmental concern shows no significant effect, highlighting the decoupling between environmental awareness and actual digital adoption behavior. Furthermore, although price discounts emerged as the strongest driver of purchase intention, heightened food safety concerns paradoxically stimulated purchases. This counterintuitive result demonstrates how a reputable retailer’s established credibility effectively mitigates consumer fears. Practically, this study provides actionable guidelines for retailers to optimize digital clearance strategies: retailers should prioritize real-time inventory transparency and economic incentives, while strategically leveraging their institutional credibility within the app to overcome consumer safety concerns regarding high-risk foods.

## 1. Introduction

The international community faces the contradictory issues of severe food shortages and excessive food waste [1], which generate significant global economic, environmental, and social problems. Economically, global food waste at the consumption stage exceeds USD 400 billion per year, and methane produced in landfills intensifies global warming [2]. In response, the United Nations emphasizes the urgent need for sustainable consumption. Specifically, Target 12.3 of the Sustainable Development Goals (SDGs) explicitly mandates halving per capita global food waste at the retail and consumer levels by 2030.

Retail enterprises have actively adopted digital technologies, such as mobile apps, to promote selling near-expired products, aiming to address food waste. Supermarkets in Taiwan have widely implemented discount promotions and real-time map features, enabling consumers to quickly access information regarding near-expiration products, locations, and quantities. While these technological systems are effective for low-risk categories such as bakery items and produce, research on “high-risk” perishables remains scarce. In the context of this study, “near-expired meat” refers specifically to fresh, unprocessed raw meat (e.g., beef, pork, and poultry) that is within one to two days of its expiration date. Due to strict storage requirements and short shelf lives, these products pose greater food safety challenges. In Taiwan, supermarkets typically apply distinct time-based price discount labels (e.g., 20% to 40% off) on these products during their final hours to accelerate clearance. Consequently, studying consumer behaviors concerning near-expired fresh meat products has significant practical and academic value.

While environmental concern often drives sustainable intentions, a well-documented “value–intention gap” frequently occurs, wherein consumers’ strong environmental awareness fails to translate into actual purchasing behavior. Furthermore, purchasing near-expired meat inherently involves high perceived health risks. The previous literature indicates that retailer credibility can serve as a powerful risk reliever, sometimes even outweighing economic incentives in driving suboptimal food purchases. However, how digital app engagement, economic incentives, and intrinsic food safety concerns interact within a highly trusted retail environment remains under-explored.

To comprehensively explain consumers’ complex behavioral intentions when shopping for high-risk, near-expired meat products via digital platforms, this study integrates two mainstream behavioral theories. The technology acceptance model’s core variables—perceived usefulness and perceived ease of use—are essential for exploring initial consumer attitudes and usage intentions toward newly launched mobile apps [3,4]. Additionally, the theory of planned behavior provides a comprehensive framework for explaining overall behavioral intentions through attitude, subjective norms, and perceived behavioral control [1,5]. Furthermore, this study incorporates three key external factors that are critical to this unique consumption context: price discounts, food safety concerns, and environmental concerns.

Recent studies have extensively explored consumer behavior regarding suboptimal foods, particularly near-expired foods, primarily focusing on low-risk categories such as visually imperfect produce or bakery items [6,7,8,9]. However, purchasing near-expired fresh meat introduces a distinct psychological barrier due to its higher perishability. To address this gap, the current study integrates the technology acceptance model and the theory of planned behavior to explore this unique context. Specifically, this research aims to address the following research questions (RQs): (RQ1) How do perceived usefulness and ease of use drive the adoption of near-expired food apps? (RQ2) To what extent do environmental concerns translate into digital app adoption? and (RQ3) How do economic incentives and food safety concerns interact to shape the final purchase intention of high-risk perishables?

This study employs an integrated framework to explore consumer purchasing intentions toward near-expired meat products. Unlike previous studies, this research explicitly investigates how price discounts and food safety concerns interact within an established retail context to influence decision-making. Specifically, this study aims to (1) determine the roles of perceived usefulness and ease of use in mobile app adoption, (2) examine the “value–intention gap” regarding environmental concerns, and (3) verify whether app usage intention translates into actual meat purchasing behavior. This study’s findings can help retailers develop actionable strategies to optimize digital tools to reduce meat waste, thereby advancing sustainable consumption.

## 2. Literature Review and Hypothesis Development

This study aims to identify the key factors influencing consumer intention to use near-expired food mobile apps and their subsequent purchasing intentions for near-expired meat products. This section reviews mainstream behavioral theories and examines the relevant literature on specific external factors critical in the context of near-expired food consumption—namely, environmental concerns, food safety concerns, and price discounts—to establish a robust theoretical model.

### 2.1. Theory of Planned Behavior

Ajzen [10] developed the theory of planned behavior as an extension of the theory of reasoned action to explain and predict human behavioral intentions [5]. The theory posits that three core factors—attitude, subjective norms, and perceived behavioral control—collaboratively influence an individual’s behavioral intentions, thereby affecting their actual behavior. Research across various contexts has consistently shown that these three factors exert a significant positive impact on consumers’ intention to use digital tools and sustainable services [1,11].

#### 2.1.1. Attitude

Regarding technology adoption and sustainability, a system’s positive evaluation significantly enhances the intention to use it [12]. This positive influence on behavioral intention has been extensively verified in relevant studies on leftover food sharing [1], environmental behavior [11], and artificial intelligence (AI) tool adoption [13]. Therefore, this study posits the following hypothesis:

**H1.**  *Usage attitude positively influences usage intention toward the near-expired food app.*

#### 2.1.2. Subjective Norms

Subjective norms refer to perceived social pressure that may influence an individual’s decision to engage in a specific behavior [5,14]. A stronger positive subjective norm motivates individuals to engage in a specific behavior. This relationship has been confirmed in contexts such as leftover food sharing [1] and general environmental behavior [11]. As such, this study proposes the following hypothesis:

**H2.**  *Subjective norms positively influence usage intention toward the near-expired food app.*

#### 2.1.3. Perceived Behavioral Control (PBC)

Perceived behavioral control is the degree of ease or difficulty an individual perceives when attempting to perform a specific behavior, reflecting their assessment of the necessary resources and opportunities [5,15]. Past research on sharing leftover food [1] and anti-consumption behavior [16] supports the significant positive impact of perceived behavioral control on behavioral intention. Therefore, this study posits the following hypothesis:

**H3.**  *Perceived behavioral control positively influences usage intention toward the near-expired food app.*

### 2.2. Technology Acceptance Model

Relying solely on the theory of planned behavior to explain people’s acceptance of new technologies may be limited, as it may not capture the antecedents that influence individual attitudes [17]. Therefore, this study integrates it with the technology acceptance model [3] to create a more comprehensive framework for evaluating consumers’ intentions to adopt newly launched mobile apps [4,18,19].

#### 2.2.1. Perceived Usefulness

Perceived usefulness is the degree to which a person believes that using a specific system enhances their performance or efficiency [3]. If users find a technology useful, they tend to develop a positive attitude toward its use [12,20]. This has been supported by research on green products [21] and technology associated with upcycled foods [22]. Therefore, this study proposes the following:

**H4.**  *Perceived usefulness positively influences usage attitude toward the near-expired food app.*

#### 2.2.2. Perceived Ease of Use

Reducing the operational burden of technology allows users to focus on tasks, thereby enhancing both perceived usefulness [18,23] and usage attitude [20]. Recent studies on green product consumption [21] confirm that a user-friendly interface is crucial for fostering positive consumer perceptions. Consequently, the following hypotheses are proposed:

**H5.**  *Perceived ease of use positively influences perceived usefulness of the near-expired food app.*

**H6.**  *Perceived ease of use positively influences usage attitude toward the near-expired food app.*

### 2.3. Environmental Concern

Environmental concern is defined as the extent of an individual’s worry about environmental issues, reflecting their sense of environmental responsibility [24]. It is often considered an important antecedent of consumers’ environmentally friendly behavior and product attitudes [15]. Environmental concern positively influences attitudes toward sharing leftover food [1] and organic foods [25]. This study explored its role as a precursor influencing attitudes toward near-expired food apps.

**H7.**  *Environmental concerns positively influence usage attitude toward the near-expired food app.*

### 2.4. Food Safety Concerns and Purchase Intention

Food safety is a critical determinant in food purchasing decisions [26]. Food safety concerns reflect consumer anxiety regarding potential health risks, such as deterioration or contamination [27]. When discussing perishable products like near-expired fresh meat, it is important to distinguish between actual food safety risks (e.g., objective hazards such as actual bacterial contamination or microbial growth due to limited shelf life) and perceived food safety risks (e.g., the consumer’s subjective anxiety and fear regarding these potential health hazards). Because fresh meat requires stringent storage temperature controls, it is generally categorized as a high-risk perishable product, elevating consumers’ perceived risks compared to other suboptimal foods like bakery items. While high perceived food safety concerns typically deter the consumption of risky foods, the previous literature indicates that retailer credibility and brand reputation can serve as powerful risk relievers [28,29]. When purchasing high-risk perishables, consumers often rely on established retail channels with transparent labeling to mitigate perceived health risks [30]. Therefore, within a highly trusted retail environment like Taiwan’s leading supermarket, heightened safety concerns may paradoxically motivate consumers to purchase near-expired meat from this specific reputable retailer rather than avoiding it entirely. Theoretically, this mechanism implies a potential moderation effect, where the retailer’s established credibility acts as a critical moderating variable that buffers, or even reverses, the traditional negative impact of food safety concerns on purchase intention.

**H8.**  *Food safety concerns positively influence purchase intention for near-expired meat products.*

### 2.5. Price Discounts

Price discounts are an important promotional instrument that businesses use to stimulate consumer purchases by enhancing product value and providing short-term monetary incentives [31,32]. Price is a crucial determinant of product purchases, and retailers’ discount strategies are known to significantly increase purchase intentions [33]. Specific discount patterns in price promotion contexts significantly affect suboptimal food purchase intentions [21]. Given that discounts enhance product value, this study proposes the following:

**H9.**  *Price discounts positively influence purchase intention for near-expired meat products.*

### 2.6. The Link Between Usage and Purchase Intention

Within the theory of planned behavior, behavioral intention is recognized as the most immediate and critical antecedent of actual behavior [5]. In the evolving digital retail landscape, this theoretical link manifests as a transition from digital engagement to physical purchasing [34,35]. For consumers, the app provides essential real-time inventory and discount information; therefore, proactive engagement with the digital platform is hypothesized to directly facilitate decision-making when purchasing high-risk perishable items in a physical store. Based on this logical progression, the following hypothesis is proposed:

**H10.**  *Usage intention toward the near-expired food app positively influences purchase intention for near-expired meat products.*

## 3. Materials and Methods

This section outlines the research framework, instrumentation, data collection procedures, and analytical techniques used to test hypotheses regarding consumers’ purchasing intentions for near-expired meat products.

### 3.1. Research Framework and Model Development

**Figure 1:**
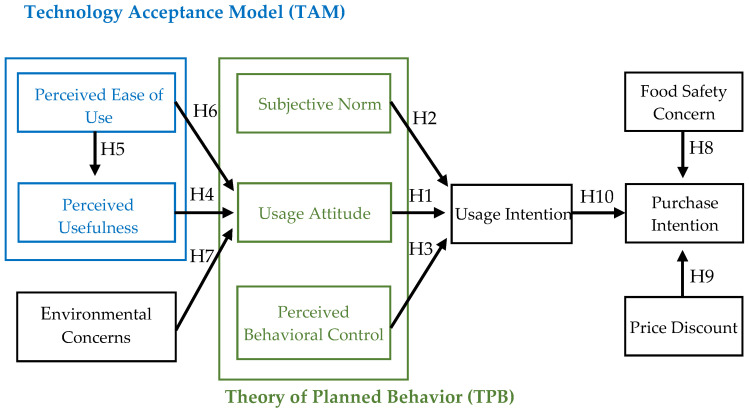
Research framework.

This study’s theoretical foundation uses an integrated structural model combining the technology acceptance model and the theory of planned behavior. Three crucial external variables—environmental concerns, food safety concerns, and price discounts—were incorporated to ensure the model reflects the unique consumption environment of near-expired products. Figure 1 illustrates the overall theoretical framework of this study.

### 3.2. Measurement Instruments

The study used a paper-based questionnaire for data collection, comprising 11 sections, with 10 latent construct variables and 1 section for personal demographic information. All construct variables were measured on a 0–10-point Likert scale. On this scale, 0 represented “strongly disagree,” 5 represented “no opinion,” and 10 represented “strongly agree.” An 11-point Likert scale (0–10) was deliberately chosen over traditional 5- or 7-point scales to provide higher measurement sensitivity and variance. This broader scale allows respondents to express their psychological attitudes and behavioral intentions with greater precision, thereby reducing data skewness and enhancing the structural model’s predictive validity.

To ensure content validity, the measurement items were adapted from established international academic scales and modified to fit the specific context of purchasing near-expired meat products. The core constructs of the technology acceptance model—specifically, perceived usefulness and perceived ease of use—were adapted from [3]. The constructs related to the theory of planned behavior—including attitude, subjective norms, and perceived behavioral control—were derived from [11,14,16].

Regarding the external variables, environmental concern was adapted from [15], whereas food safety concern was based on the scale in [27]. Items for price discounts and purchase intention were adapted from [21,33]. Finally, usage intention was measured using items adapted from [19,36].

### 3.3. Sampling and Data Collection

This study selected PX Mart, Taiwan’s leading supermarket chain, as the research context due to its high market penetration and established brand recognition. To address food waste, PX Mart launched a new “Time-Saving Map” feature on its mobile app in late 2024, providing real-time inquiry services for various near-expired food products, including meat. This study specifically focused on near-expired meat products, categorized as high-risk perishables with significant food waste implications due to their stringent storage requirements and limited shelf life. To maintain geographic consistency, the sampling scope was defined to include PX Mart retail outlets in Taichung City, Taiwan.

Given the extensive size of the target population and the logistical impracticality of obtaining a comprehensive sampling frame, this study used a nonprobability convenience sampling approach. Fieldwork was executed through personal investigations conducted between February and April 2025. To ensure contextual clarity and mitigate response bias, survey personnel provided standardized on-site demonstrations of the mobile app’s functional interface (e.g., the “Time-Saving Map”). This procedure ensured that even respondents unfamiliar with the digital platform could provide informed responses regarding their usage and purchase intentions.

Regarding the sample metrics, the survey was administered in-person to 494 individuals, yielding 494 initially retrieved questionnaires. Following a rigorous screening process to exclude incomplete responses or those exhibiting inconsistent patterns, 418 valid questionnaires were retained for the final analysis. This process resulted in an effective recovery rate of 84.62%, providing a robust empirical foundation for testing the proposed theoretical framework.

### 3.4. Data Analysis

This study employed SPSS version 18.0 (IBM Corp., Armonk, NY, USA) for descriptive statistical analysis and SmartPLS version 4.0 (SmartPLS GmbH, Oststeinbek, Germany) for partial least squares structural equation modeling (PLS-SEM) to test the hypotheses. The analysis proceeded in two stages. First, the measurement model was assessed for internal consistency reliability (Cronbach’s α and composite reliability), convergent validity (factor loadings and average variance extracted), and discriminant validity (Fornell–Larcker criterion). Second, the structural model was evaluated using bootstrapping with 5000 subsamples to test the significance of path coefficients, the coefficient of determination (R^2^), and overall goodness-of-fit.

To explicitly formulate the connections between the constructs based on the research framework (Figure 1), the inner structural model is represented by the following mathematical equations:

(1) *PU* = *β*_5_ *PEOU* + *ζ*_1_;
(2) *ATT* = *β*_4_ *PU* + *β*_6_ *PEOU* + *β*_7_ *EC* + *ζ*_2_;
(3) *UI* = *β*_1_ *ATT* + *β*_2_ *SN* + *β*_3_ *PBC* + *ζ*_3_;
(4) *PI* = *β*_10_ *UI* + *β*_8_ *FSC* + *β*_9_ *PD* + *ζ*_4_.

In these equations, the construct indicators are defined as follows: *PU* (perceived usefulness), *PEOU* (perceived ease of use), *ATT* (usage attitude), EC (environmental concerns), *UI* (usage intention), *SN* (subjective norm), *PBC* (perceived behavioral control), *PI* (purchase intention), *FSC* (food safety concern), and *PD* (price discount). The *βn* coefficients represent the standardized path weights corresponding to each formulated hypothesis (*H_n_*), indicating the strength and direction of the respective relationships. The *ζ* terms denote the structural error (residual variance) that is not explained by the predictors in each respective equation.

Additionally, a post hoc power analysis was conducted using the G*Power version 3.1.4 software (Heinrich-Heine-Universität Düsseldorf, Düsseldorf, Germany). Given the sample size of 418, an alpha level of 0.05, a medium effect size (*f*^2^ = 0.15), and a maximum of four predictors for the endogenous construct, the statistical power exceeded 0.99. This value was well above the recommended threshold of 0.80, confirming that the sample size was highly adequate for the PLS-SEM analysis.

## 4. Results

This section sequentially reports the sample analysis, the assessment of the measurement model (reliability and validity), the descriptive statistics of the constructs, and the results of the structural equation modeling used to test the research hypotheses.

### 4.1. Sample Analysis

A total of 418 valid questionnaires were analyzed. Table 1 presents the demographic profile, indicating that the majority of respondents were female (60.30%), aged 41–50 years (33.50%), and possessed a college or university degree (57.90%). Regarding economic status, 72.97% of respondents reported a monthly income of TWD 50,000 or less. This demographic distribution aligns with the typical consumer profile of the supermarket chain under study [37].

### 4.2. Measurement Model

The measurement model was rigorously evaluated using confirmatory factor analysis in SmartPLS 4.0. To establish the reliability of the research instrument, internal consistency was first assessed using Cronbach’s α. Table 2 shows that the values for all 10 latent constructs ranged from 0.922 to 0.982, significantly exceeding the established acceptable standard of 0.7 and confirming the questionnaire’s high internal consistency [38,39].

Convergent validity was subsequently assessed using three primary metrics: factor loadings, composite reliability, and average variance extracted. In the initial analysis of factor loadings, one item (J1: “I am concerned about the condition of our living environment.”) from the environmental concern dimension was excluded due to a low loading of 0.34. This deletion occurred because J1 primarily captures a passive emotional state (worry or anxiety) toward the environment, whereas the remaining items (J2–J7) measure proactive environmental responsibility, awareness, and personal relevance. This distinction likely introduced multidimensionality. However, the remaining six items (J2–J7) robustly and comprehensively capture the core domain of consumers’ actionable environmental awareness.

For the remaining 44 items, factor loadings ranged from 0.81 to 0.98, satisfying the minimum recommended standard of 0.7 [40]. Furthermore, the composite reliability values for all constructs spanned from 0.96 to 0.99, while the average variance extracted values ranged from 0.82 to 0.95. Both sets of indices comfortably surpassed their respective recommended thresholds of 0.7 and 0.5, as suggested by Hair et al. [41]. These results provide robust empirical support for the model’s convergent validity.

Discriminant validity—which confirms that the constructs are distinct—was assessed using the Fornell–Larcker criterion. The results in Table 3 confirm that the square root of the average variance extracted for each construct (the diagonal value) exceeded its correlation coefficient with any other construct, thus validating the model’s discriminant validity [41].

### 4.3. Descriptive Statistics

This section summarizes the descriptive statistics for all constructs, measured on a 0–10 point Likert scale. The results indicate that environmental concern achieved the highest overall mean (*M* = 8.55), reflecting a strong level of environmental awareness among respondents. Conversely, subjective norms yielded the lowest mean (*M* = 6.47), although it remained well above the neutral midpoint (5.00). All other constructs—perceived behavioral control (*M* = 8.06), usage attitude (*M* = 7.38), and price discounts (*M* = 7.34)—exhibited mean scores indicating generally positive attitudes. Notably, both usage intention (*M* = 7.17) and purchase intention (*M* = 7.06) showed positive inclinations, confirming that respondents are generally willing to use the mobile app to purchase near-expired meat products.

### 4.4. Structural Model Assessment

The structural model was evaluated using partial least squares structural equation modeling to examine the hypothesized relationships between constructs. This approach employed a bootstrapping procedure with 5000 resamples to determine statistical significance [42]. The model’s predictive capability was first assessed using the coefficient of determination (*R*^2^). This study followed the thresholds suggested by Hair et al. [41]: values exceeding 0.75 indicate strong explanatory power, and those around 0.50 indicate moderate power. The results demonstrated that the integrated framework possesses substantial predictive capacity; specifically, usage intention achieved an *R*^2^ of 0.783, indicating strong predictive power. In contrast, purchase intention showed a high predictive power (*R*^2^ = 0.723). Furthermore, usage attitude accounted for 62.9% of the variance, and perceived usefulness yielded an *R*^2^ of 0.493, effectively aligning with the moderate explanatory power threshold.

To complement the explanatory power analysis, the model’s overall fit was assessed using the goodness-of-fit indicator. The calculated goodness-of-fit value of 0.554 significantly exceeded the established threshold of 0.36 for a strong fit [43], confirming that the proposed model possesses excellent goodness-of-fit. Collectively, these findings validate that the integrated theoretical framework provides a robust basis for predicting consumer purchase intention toward near-expired meat products.

### 4.5. Hypotheses Testing Results

**Figure 2:**
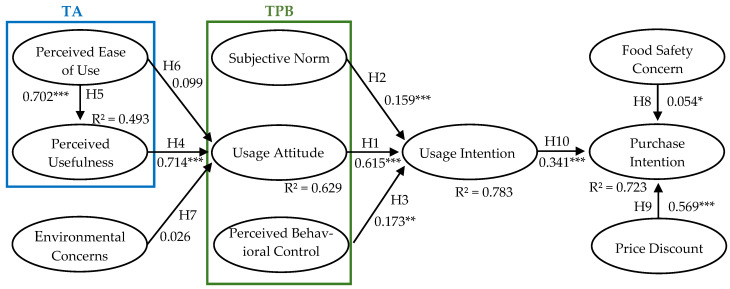
Analysis results of the structural model. Path coefficients are presented as standardized estimates (*β*). * *p* < 0.05, ** *p* < 0.01, *** *p* < 0.001.

Table 4 and Figure 2 summarize the path coefficients, significance levels, and the coefficient of determination (*R*^2^) of the structural model, and the empirical results support 8 of the 10 proposed hypotheses. Within the theory of planned behavior framework, usage attitude (β = 0.62), subjective norms (β = 0.16), and perceived behavioral control (*β* = 0.17) each exerted a significant positive influence on usage intention, thereby supporting H1, H2, and H3. Regarding technology acceptance constructs, perceived usefulness significantly influenced usage attitude (*β* = 0.71, *p* < 0.01; H4 is supported), whereas perceived ease of use was a strong predictor of perceived usefulness (*β* = 0.70; H5 is supported). However, perceived ease of use did not have a significant direct impact on usage attitude (*β* = 0.10, *p* > 0.05; H6 is not supported), and similarly, the external variable of environmental concern did not significantly influence attitude (*β* = 0.03, *p* > 0.05; H7 is not supported).

Regarding the determinants of purchase intention, price discounts emerged as the most potent predictor (*β* = 0.57, *p* < 0.01; H9 is supported), followed by usage intention (*β* = 0.34, *p* < 0.01; H10 is supported). Notably, food safety concerns also demonstrated a significant positive, although comparatively weaker, influence on purchase intention (*β* = 0.05, *p* < 0.01; H8 is supported).

The mediation analysis revealed significant indirect effects, further elucidating the model’s complex dynamics. Specifically, perceived usefulness and perceived ease of use indirectly influenced usage intention with coefficients of 0.44 and 0.37, respectively. Furthermore, these technological factors indirectly affected the ultimate purchase intention through the mediating mechanism of usage intention. This finding confirms that the mobile app’s utility constitutes a critical pathway that bridges initial technology acceptance with actual near-expired meat purchasing behavior in a physical retail context.

## 5. Discussion

This study aimed to decode the complex consumer decision-making process regarding high-risk, near-expired fresh meat within a digital retail context. By integrating the technology acceptance model and the theory of planned behavior with external variables, this study successfully addressed its primary objectives. First, it determined that perceived usefulness, rather than mere ease of use, is the primary driver of mobile app adoption. Second, it confirmed the existence of an “attitude–behavior gap,” where high environmental concerns fail to translate into digital adoption. Finally, it verified that digital usage intention robustly translates into actual physical purchasing behavior, driven predominantly by price discounts but paradoxically supported by food safety concerns within a reputable retail setting.

### 5.1. Theoretical Contributions

#### 5.1.1. The Dominance of Utility in Technology Acceptance

In the context of mature digital retail environments, the practical utility of an application significantly outweighs its interface simplicity in driving user attitude. The empirical results demonstrated that perceived usefulness exerted the strongest positive influence on usage attitude (*β* = 0.71, *p* < 0.01). Conversely, the direct influence of perceived ease of use on attitude was statistically insignificant (*β* = 0.10, *p* > 0.05). This divergence implies that in an era where mobile app accessibility is a baseline consumer expectation, a user-friendly interface is merely a foundational prerequisite. As supported by the recent technology adoption literature [12,18], the true driver of positive consumer evaluation is the functional value derived from real-time and accurate inventory information. Therefore, this study refines the technology acceptance model for modern retail, confirming that functional utility bridges the gap between initial technology trials and sustained usage attitude.

#### 5.1.2. Decoupling of Environmental Concerns

High environmental awareness does not automatically translate into adopting digital tools designed for sustainable consumption. Despite respondents demonstrating an exceptionally high level of environmental awareness (achieving the highest mean score of 8.55), environmental concern did not significantly influence usage attitude (*β* = 0.03, *p* > 0.05). This finding suggests a potential boundary condition for the traditional premise in the sustainability literature, which generally posits environmental concern as a universal antecedent of pro-environmental behavioral attitudes [1,15]. While studies on general organic food or green products often find a strong link between environmental concern and purchase attitude, our results indicate that in the specific context of high-risk near-expired meat, consumers appear to prioritize immediate personal benefits over abstract environmental values. This observation aligns with the “value–intention gap” frequently discussed in digital food waste reduction initiatives [44], suggesting that appealing solely to environmental values might be insufficient to drive the adoption of sustainable digital tools.

#### 5.1.3. Economic Primacy and the Trust Paradox

While economic incentives predominantly drive purchasing near-expired meat, heightened food safety concerns paradoxically stimulate, rather than deter, purchase intentions when facilitated by an established retailer. Price discounts emerged as the most potent predictor of purchase intention (*β* = 0.57, *p* < 0.01). Simultaneously, food safety concerns demonstrated a positive influence on purchase intention (*β* = 0.05, *p* < 0.01). Prior studies have often argued that brand loyalty or retailer reputation can be a stronger predictor of high-risk or organic food purchases than price discounts [28,30]. However, our findings indicate that in a mobile app environment specifically designed for clearance sales, economic incentives remain paramount, which aligns with recent studies on suboptimal food promotions [21]. However, the positive influence of food safety concerns reveals a unique “trust paradox”: instead of avoiding high-risk perishable foods entirely, safety-conscious consumers migrate toward highly trusted, established supermarket channels to mitigate perceived health risks [29]. This highlights a dual pathway where extreme price sensitivity is balanced by a deep reliance on the established credibility of the retailer, presenting a novel contribution to the literature on suboptimal food consumption.

### 5.2. Managerial Implications

Our findings offer critical, evidence-based strategies for retailers aiming to optimize selling near-expired high-risk foods. First, retailers must recognize the absolute primacy of economic incentives. Since price discounts had the strongest impact on purchase intention, supermarkets should utilize mobile app push notifications to broadcast real-time, targeted discounts during peak clearance hours.

Second, to fully leverage the “trust paradox,” retailers must actively reinforce their institutional credibility to overcome consumers’ inherent food safety concerns. Retailers should enhance product traceability within the mobile app—clearly displaying expiration timeframes, strict storage temperature logs, and safety certifications. By transforming the app from a mere discount locator into a transparent information hub, retailers can effectively mitigate the perceived risks associated with fresh meat.

Finally, addressing the “attitude–behavior gap” requires innovative engagement strategies. Although abstract environmental values failed to drive adoption, apps can incorporate gamified feedback loops—such as displaying “You saved X kg of carbon emissions today” or offering sustainability badges. As suggested by recent digital marketing trends, gamifying the retail experience can transform abstract environmental concepts into tangible personal achievements, thereby subtly aligning economic motivations with sustainable development goals.

### 5.3. Limitations and Future Research

This study has several limitations that provide avenues for future research. First, the sample was restricted to consumers of a single leading supermarket chain in Taiwan, which may limit the generalizability of the findings. Consumer perceptions of food safety, waste, and discounted products vary considerably across cultural contexts. In Taiwan, the high density of supermarkets has cultivated a consumer culture that prioritizes convenience and places a relatively high degree of trust in established corporate retail brands. Consequently, the “trust paradox” observed in this study—where safety-conscious consumers actively purchase near-expired meat through a trusted app—may be particularly pronounced in regions with highly organized retail infrastructure. Future studies should expand the geographic and cultural scope to explore whether these findings hold in different cultural settings, where public trust in food supply chains may vary. Second, while this study confirms that price discounts are the major driver of behavior, it did not measure the specific discount thresholds. Future experimental research should focus on varying levels of discounts (e.g., 20% vs. 50% off) to determine the optimal pricing strategy that triggers purchases without eroding brand equity. Finally, given the counterintuitive positive relationship between food safety concerns and purchase intention within a reputable retail setting, future structural models should explicitly incorporate “retailer credibility” or “brand trust” as moderating variables to fully understand the mechanics of the trust paradox.

## 6. Conclusions

This study successfully integrates the technology acceptance model and the theory of planned behavior to elucidate consumer purchasing decisions regarding high-risk, near-expired meat in a digital retail setting. The findings highlight that the functional utility of mobile applications and strategic price discounts are the primary drivers of sustainable consumer behavior, significantly outweighing abstract environmental concerns. Furthermore, the established credibility of reputable retailers plays a crucial role in mitigating food safety concerns, creating a “trust paradox” that encourages purchasing suboptimal perishables. These insights provide valuable guidelines for retailers to optimize digital clearance strategies and actively contribute to global food waste reduction goals.

## Figures and Tables

**Table 1:** Personal demographic information of the respondents.

Variable	Category	*N*	%
Gender	Male	166	39.70%
	Female	252	60.30%
Age	20 years and below (inclusive)	14	3.30%
	21–30 years	101	24.20%
	31–40 years	92	22.00%
	41–50 years	140	33.50%
	51–60 years	54	12.90%
	61 years (inclusive)	17	4.10%
Education	Level below junior high school (inclusive)	16	3.80%
	High school (vocational)	90	21.50%
	College or university	242	57.90%
	Master’s	63	15.10%
	Doctorate	7	1.70%
Personal Monthly Income	Below TWD 28,590	71	16.99%
	TWD 28,591–35,000	96	22.97%
	TWD 35,001–50,000	138	33.01%
	TWD 50,001–80,000	74	17.70%
	TWD 80,001–110,000	22	5.26%
	TWD 110,001–220,000	12	2.87%
	Above TWD 220,001	5	1.20%

**Table 2:** Revised estimated parameters for the measurement model.

Dimension	Item	Factor Loading	S.E.	t-Value	*p*-Value	CR	AVE
Purchase Intention (PI)	A1: I am very likely to purchase the near-expired meat with a 40% discount on the PX Mart app.	0.92	0.01	75.35	0.00	0.96	0.86
	A2: I am willing to purchase the near-expired meat with a 40% discount on the PX Mart app.	0.96	0.01	143.01	0.00		
	A3: In the future, I am willing to go to PX Mart to purchase the near-expired meat with a 40% discount featured on the app.	0.96	0.01	146.49	0.00		
	A4: I am willing to recommend the near-expired meat with a 40% discount on the PX Mart app to my family and friends.	0.87	0.02	48.36	0.00		
Usage Intention (UI)	B1: I am willing to use PX Mart’s near-expired food app in the future.	0.94	0.01	125.84	0.00	0.96	0.87
	B2: I will use PX Mart’s near-expired food app whenever I have the need in the future.	0.94	0.01	114.85	0.00		
	B3: I will recommend PX Mart’s near-expired food app to my relatives and friends in the future.	0.90	0.02	55.07	0.00		
	B4: Overall, I think PX Mart’s near-expired food app is worth using.	0.93	0.01	99.98	0.00		
Usage Attitude (ATT)	C1: Overall, using PX Mart’s near-expired food app meets my needs.	0.90	0.01	68.58	0.00	0.967	0.86
	C2: I think using PX Mart’s near-expired food app is a good idea.	0.94	0.01	90.18	0.00		
	C3: Overall, PX Mart’s near-expired food app is worthwhile to use.	0.95	0.01	134.11	0.00		
	C4: To me, using PX Mart’s near-expired food app is a pleasant experience.	0.92	0.01	79.81	0.00		
	C5: I think it is wise to use PX Mart’s near-expired food app.	0.92	0.01	81.10	0.00		
Subjective Norm (SN)	D1: People who are important to me (family, partner, friends…) think it is good for me to use PX Mart’s near-expired food app.	0.98	0.00	288.90	0.00	0.98	0.93
	D2: People who are important to me (family, partner, friends…) approve of my use of PX Mart’s near-expired food app.	0.97	0.01	161.96	0.00		
	D3: Most people who are important to me (family, partner, friends…) want me to use PX Mart’s near-expired food app.	0.95	0.01	119.64	0.00		
Perceived behavioral control (PBC)	E1: For me, using PX Mart’s near-expired food app is possible.	0.88	0.02	50.85	0.00	0.96	0.84
	E2: If I want to, I am able to use PX Mart’s near-expired food app.	0.92	0.01	66.30	0.00		
	E3: Whether or not I use PX Mart’s near-expired food app is entirely up to my own decision.	0.93	0.01	66.93	0.00		
	E4: As long as I am willing, using PX Mart’s near-expired food app is easy.	0.94	0.01	71.91	0.00		
	E5: The decision to use PX Mart’s near-expired food app is completely under my control.	0.91	0.02	52.74	0.00		
Food safety concerns (FSC)	F1: I am concerned about the quality and safety of the meat currently on the market.	0.91	0.07	13.02	0.00	0.97	0.88
	F2: I worry about whether the meat on the market contains residual growth hormones.	0.96	0.08	11.57	0.00		
	F3: I worry about whether the meat on the market contains residual antibiotics.	0.96	0.08	11.61	0.00		
	F4: I worry about whether the meat on the market is contaminated with bacteria.	0.91	0.07	12.42	0.00		
Price Discount (PD)	G1: The 40% off promotion for near-expired meat at PX Mart is attractive to me.	0.98	0.00	236.47	0.00	0.99	0.95
	G2: The 40% off promotion for near-expired meat at PX Mart makes me feel like I am getting good value for money.	0.97	0.01	165.66	0.00		
	G3: The 40% off promotion for near-expired meat at PX Mart is valuable to me.	0.98	0.00	339.35	0.00		
	G4: I am interested in the 40% off promotion for near-expired meat at PX Mart.	0.97	0.01	190.14	0.00		
Perceived Usefulness (PU)	H1: I think PX Mart’s near-expired food app provides me with useful information.	0.92	0.01	73.21	0.00	0.96	0.85
	H2: I think using PX Mart’s near-expired food app helps me save shopping time.	0.90	0.02	62.08	0.00		
	H3: I think PX Mart’s near-expired food app satisfies my shopping needs.	0.94	0.01	130.88	0.00		
	H4: Overall, I think PX Mart’s near-expired food app is helpful to me.	0.93	0.01	79.66	0.00		
Perceived ease of use (PEOU)	I1: I think operating PX Mart’s near-expired food app is simple and easy.	0.94	0.01	79.85	0.00	0.98	0.87
	I2: I think the user interface of PX Mart’s near-expired food app is friendly.	0.95	0.01	98.22	0.00		
	I3: I think the way to use PX Mart’s near-expired food app is not confusing.	0.95	0.01	101.74	0.00		
	I4: I think it is easy to become skillful at using PX Mart’s near-expired food app.	0.95	0.01	113.45	0.00		
	I5: I think the information provided by PX Mart’s near-expired food app is clear and understandable.	0.94	0.01	71.74	0.00		
	I6: I think by using PX Mart’s near-expired food app, I can easily buy the near-expired meat I need.	0.87	0.02	35.91	0.00		
Environmental Concern (EC)	J2: I believe everyone has a responsibility to protect the environment we live in.	0.88	0.04	25.31	0.00	0.96	0.82
	J3: Environmental sustainability is an important consideration when I choose products.	0.81	0.03	25.76	0.00		
	J4: To me, protecting the environment we live in is important.	0.92	0.02	42.71	0.00		
	J5: I think the issue of resource waste is closely related to everyone.	0.91	0.02	48.17	0.00		
	J6: I think the problems regarding our living environment cannot be ignored.	0.95	0.01	102.00	0.00		
	J7: I think we should care more about the problems of our living environment.	0.94	0.01	87.11	0.00		

Note: CR = composite reliability; AVE = average variance extracted. A CR value greater than 0.7 indicates excellent internal consistency reliability, while an AVE value greater than 0.5 demonstrates adequate convergent validity.

**Table 3:** Validity of each facet after correction.

Dimension	1	2	3	4	5	6	7	8	9	10
1. Subjective Norm	**0.97**									
2. Usage Intention	0.74	**0.93**								
3. Usage Attitude	0.75	0.87	**0.93**							
4. Price Discount	0.61	0.70	0.70	**0.98**						
5. Environmental Concern	0.19	0.18	0.21	0.15	**0.90**					
6. Perceived Ease of Use	0.53	0.56	0.61	0.53	0.29	**0.93**				
7. Perceived Usefulness	0.72	0.77	0.79	0.72	0.22	0.70	**0.92**			
8. Perceived Behavioral Control	0.65	0.76	0.78	0.62	0.27	0.66	0.80	**0.92**		
9. Purchase Intention	0.63	0.75	0.68	0.81	0.14	0.52	0.67	0.64	**0.93**	
10. Food Safety Concern	0.10	0.07	0.07	0.07	0.33	0.13	0.07	0.07	0.12	**0.94**

Note: The bold diagonal values are the square roots of the AVE, and the nondiagonal values are the correlation coefficients between the facets.

**Table 4:** Path coefficients between the constructs.

Hypothesis	Path Relationship	Standardized Coefficient	Standard Deviation	t-Value	*p*-Value	Hypothesis Result
H1	Usage Attitude → Usage Intention	0.62	0.07	9.36	< 0.001 ***	Supported
H2	Subjective Norm → Usage Intention	0.16	0.04	3.83	< 0.001 ***	Supported
H3	PBC → Usage Intention	0.17	0.06	3.12	< 0.01 **	Supported
H4	Perceived Usefulness → Usage Attitude	0.71	0.05	16.02	< 0.001 ***	Supported
H5	Perceived Ease of Use → Perceived Usefulness	0.70	0.04	18.42	< 0.001 ***	Supported
H6	Perceived Ease of Use → Usage Attitude	0.10	0.06	1.61	0.11	Not Supported
H7	Environmental Concerns → Usage Attitude	0.03	0.04	0.69	0.49	Not Supported
H8	Food Safety Concern → Purchase Intention	0.05	0.02	2.21	0.027 *	Supported
H9	Price Discount → Purchase Intention	0.57	0.06	9.56	< 0.001 ***	Supported
H10	Usage Intention → Purchase Intention	0.34	0.06	5.50	< 0.001 ***	Supported

Note: PBC = perceived behavioral control. * *p* < 0.05, ** *p* < 0.01, *** *p* < 0.001.

## Data Availability

The data supporting the findings of this study are not publicly available because the dataset includes personal data from participants who consented under the conditions of confidentiality and non-disclosure, and we are obliged to uphold their privacy rights. However, the data are available from the corresponding author, H.-S.C., upon reasonable request and with respect to the aforementioned constraints.
